# Foot length, chest and head circumference measurements in detection of Low birth weight neonates in Mekelle, Ethiopia: a hospital based cross sectional study

**DOI:** 10.1186/s12887-017-0866-0

**Published:** 2017-04-21

**Authors:** Marta Yemane Hadush, Amanuel Hadgu Berhe, Araya Abrha Medhanyie

**Affiliations:** 1 School of Medicine, Mekelle Univesity, College of Health Sciences, Mekelle, Ethiopia; 2 School of Public Health, Mekelle University, College of Health Sciences, Mekelle, Ethiopia

**Keywords:** Low birth weight, Anthropometry, Chest circumference, Head circumference, Foot length, Surrogate

## Abstract

**Background:**

Low birth weight (Birth weight < 2500 g) is a leading cause of prenatal and neonatal deaths. The early identification of Low birth weight (LBW) neonates is essential for any comprehensive initiative to improve their chance of survival. However, a large proportion of births in developing countries take place at home and birth weight statistics are not available. Therefore, there is a need to develop simple, inexpensive and practical methods to identify low birth weight (LBW) neonates soon after birth.

**Methods:**

This is a hospital based cross sectional study. Four hundred twenty two (422) live born neonates were included and anthropometric measurements were carried out within 24 h of birth by three trained nurses. Birth weight was measured by digital scale. Head and chest circumference were measured by using non extendable measuring tape and foot length with hard transparent plastic ruler. Data was entered into SPSS version 20 for analysis. Characteristics of study participants were analyzed using descriptive statistics such as frequency and percentage for categorical data and mean and standard deviation for continuous data. Correlation with birth weight using Pearson’s correlation coefficient and linear regression were used to identify the association between dependent and independent variables. Receiver operating characteristic (ROC) curve was used to evaluate accuracy of the anthropometric measurements to predict LBW.

**Results:**

The prevalence of low birth weight was found to be 27%. All anthropometric measurements had a positive correlation with birth weight, chest circumference attaining the highest correlation with birth weight (*r* = 0.85) and foot length had the weakest correlation (*r* = 0.74). Head circumference had the highest predictive value for birth weight (AUC = 0.93) followed by Chest circumference (AUC = 0.91). A cut off point of chest circumference 30.15 cm had 84.2% sensitivity, 85.4% specificity and diagnostic accuracy (*P* < 0.001). A cut off point of head circumference 33.25 had the highest positive predictive value (77%).

**Conclusion:**

Chest circumference and head circumference were found to be better surrogate measurements to identify low birth weight neonates.

## Background

According to the World Health Organization (WHO) definition, neonates with birth weights of less than 2500g are classified as low birth weight (LBW) regardless of gestational age. Subcategories include Very low birth weight, which is less than 1500 g and extremely low birth weight, which is less than 1000 g [1].

A significant progress has been made in the reduction of child mortality in the past decades worldwide. Though the under five mortality rate has decreased globally by about 50% (from 90 to 48 deaths per 1000 live births) in the year 1990 and 2012 respectively, the neonatal mortality rate decreased only 36%, from 33 deaths/1000 live births to 21 deaths/1000 live births over the same period [2]. Globally one sixth of neonates are born low birth weight (LBW, <2500 g), which is an underlying factor for 60 to 80% of neonatal deaths [3].

The WHO country cooperation strategy 2008–2011 showed that the prevalence of low birth weight in Ethiopia was estimated to be 14%. It is one of the highest in the world [1]. Previous studies done in Ethiopia show that there is a decline in mean birth weight and that there is an increasing trend in LBW from 1970 to 1990’s. For example in south western part of the country among health institutional deliveries the incidence of LBW is 22.5% [4].

LBW is a leading cause of perinatal deaths and remains a worldwide issue and one of the most important public health problems particularly in developing countries [5].

The risk of death increases as the birth weight is lower; neonates born with weight between 2000 and 2499g are 4 times more likely to die during their first 28 days of life than neonates born with weight between 2500 and 2999g, and 10 times more likely to die than those weighing 3000–3499g [6]. Thus early identification of the LBW neonates is essential for any intervention to improve their chances of survival. Despite most of the worlds’ LBW neonates are born in developing countries, birth weight statistics are not available because significant proportion of births takes place at home. According to Ethiopian Demographic and Health Survey 2000, 2005, and 2011 the trend on neonatal mortality were 49, 39, and 37 respectively. But having this, Ethiopia has limited birth weight estimates as there are home deliveries, inaccurate weighing scales and poor documentation of birth weights [7].

Weighing scale was, is and will be the appropriate, accurate and standard for detection of neonates’ birth weight. In this study finding a surrogate is particularly intended targeting the disadvantaged communities where the access to health facilities might be difficult and where neonates are not weighed routinely due to paucity of a suitable weighing scale at the health facilities. These surrogates will help in the detection of LBW neonates and bring them to medical attention. The measurements can be done by health extension worker whose responsibilities are to do home visits and detect health problems in the community and get the people come to medical attention.

Though the baseline population in Ethiopia is similar with WHO standard (uses WHO growth charts), for the aforementioned reasons there is a need to develop simple to use and carry, inexpensive and practical methods to identify LBW neonates soon after birth. This will be useful in early identification and prompt referral. Efforts should be made to find an effective method for identification of these high-risk neonates in the community.

A number of alternative anthropometric measurements have been proposed as surrogate for birth-weight. These include the circumferences of the neonates’ head, chest, and mid arm [8–14]. In most studies done so far chest circumference has the highest sensitivity followed by head circumference, mid upper arm circumference (MUAC) and foot length [10] on identifying LBW.

Chest and head circumference were used to identify LBW preterm neonates in Ethiopia, and the cut-off points with the best sensitivity and specificity identified were 30 cm and 31 cm for chest and head circumference respectively [11].

Though in most of the previous studies chest circumference has a good sensitivity, the cut off points varies among the studies. This study aims to find a better surrogate anthropometric measurement to detect LBW neonates and will be a supplement to the previously done studies.

## Methods

### Study setting

The study was conducted in Ayder Referral hospital (ARH), which is located in Mekelle city, Tigray region, north Ethiopia which is found at 783 km from Addis Ababa, the capital city of Ethiopia. The Hospital provides a broad range of medical services to both in and out patients of all age groups. It stands as the second largest hospital in the nation with a total capacity of about 500 inpatient beds in four major departments and other specialty units. Pediatrics and child health is one of the departments in the hospital, care for neonates being one of the services it provides. There is fairly equipped neonatal intensive care unit (NICU) and provides a tertiary level of care. About 40% of the admissions to NICU are from other health facilities in the region and the remainder 60% being from obstetrics ward in the hospital which was also the second study area. The annual rate of delivery in the hospital is 2800.

Ethical clearance was obtained from the Ethical Review Board of Mekelle University, College of Health Science. Letter of permission was presented to Ayder referral hospital. Verbal informed consent was obtained from mothers/guardian of the newborns after the purpose of the study is explained. Verbal informed consent was done for most of the participants were illiterates.

### Study design, data collection and analysis

This was a hospital based quantitative cross sectional study design. Data was collected by using data collection format by nurses who work at the NICU after being given one day training on how to measure the neonates’ head and chest circumferences by using non extendable measuring tape, with a width of 1.0 cm and subdivisions of 0.1 cm and foot length using hard transparent plastic ruler. Reliability of measurements was checked by doing repeat measurements for each test intermittently by the supervisor.

Four hundred twenty two (422) live born babies were included which was calculated using single population proportion formula. Anthropometric measurements were carried out within 24 h of birth by the trained nurses. Head circumference (HC) was measured between glabellas anteriorly and along the occipital prominence posteriorly. Chest circumference (CC) was measured at the level of nipple at the end phase of expiration. Foot length was measured from the heel to tip of the big toe using hard transparent plastic ruler. Babies were weighed naked on digital weighing scale. Weight scale was calibrated before every measurement using a material of standard weight of 1000g (bag of 1000 ml IV fluid). Gestational age of the newborns was taken from mothers’ medical record.

After the collected data was edited and checked for completeness and consistency; it was directly entered into SPSS version 20. Characteristics of study participants were analyzed using descriptive statistics such as frequency and percentage for categorical data and mean and standard deviation (SD) for continuous data.

Pearson’s correlation coefficient and linear regression analyses were done to examine linear relationship between two or more continuous variables.

Using the chi square analyses, the accuracy of all the variables in identifying LBW neonates were compared. Statistical significance was declared at probability (P) value of < 0.05 and 95% confidence interval (CI) was used to show the strength of association between independent and dependent variables.

Non-parametric receiver operating characteristic (ROC) curve analysis was carried out to calculate 95% confidence intervals of areas under the curve (AUC) that is to determine the overall accuracy and the sensitivity of the cut off points to identify best surrogate anthropometric measurement. Finally the predictive performances of the cut off points were calculated.

## Result

### Characteristics of study population

The total number of study participants enrolled in this study was 422, of which 50.24% of them were males (Table 1). The birth weights of the subjects ranged from 770 g to 4760 g, with a mean birth weight of 2807 ± 691.82 g. Using the World health assembly cut-off value of <2500 g, a total of 114 neonates (27%) were LBW. As Table 1 shows the proportion of LBW among females is found to be higher than males (57.9% vs. 42.1%).

**Table 1:** Sex distribution of the neonates based on weight categories

Weight(gm)	Male’s frequency (%)	Female’s frequency (%)	Total frequency (%)
>2500	163(53.27%)	143(46.73%)	306(100%)
<2500	48(42.1%)	66(57.9%)	114(100%)
Total	211(50.24%)	209(49.76%)	420(100%)

Table 2 demonstrates the distribution of neonates in weight and gestational age categories. It shows that 58.77% of those with LBW (<2500g) are preterm and 41.23% are term. The rate of prematurity is 18.18%.

**Table 2:** Gestational age distribution of the neonates based on weight categories

Weight (gm)	Gestational age(weeks)			Total
	>42	37 – 42	<37	
>2500	2(0.66%)	293(96.38%)	9(2.96%)	304(100%)
<2500	0	47(41.23%)	67(58.77%)	114(100%)
Total	2(0.48%)	340(81.34%)	76(18.18%)	418(100%)

### Anthropometric parameters for different weight categories

Table 3 outlines the mean values of the anthropometric variables for the different weight categories. Overall the mean for each of the measurements for LBW neonates were; 31.21 ± 2.48 for HC, 27.27 ± 3.2 cm for CC and 6.8 ± 0.85 cm for FL.

**Table 3:** Mean values of anthropometric variables for the different weight categories

Parameters	Weight (gm)	Number	Mean	Std. Deviation
Head circumference of the neonate (HC)	>2500	308	35.2977	1.67445
	<2500	114	31.2132	2.48067
Chest circumference of the neonate (CC)	>2500	308	32.3712	2.01901
	<2500	114	27.2789	3.22091
Foot length of the neonate (FL)	>2500	308	7.9340	.45403
	<2500	114	6.8097	.85747

Table 4 shows the results of the analysis of variance with respect to the three measurements; there was a statistically significant difference among the two weight categories for all three measurements. It indicates that all the anthropometric variables had significant, linear, positive correlation with birth weight (*p* < 0.001). CC attained the highest correlation with birth weight (*r* = 0.85) while FL attained the lowest (*r* = 0.746).

**Table 4:** Pearson correlation coefficient of anthropometric variables

Anthropometric	Pearson Correlation	R^2^	*P*-Value
Variables	Coefficient (r)		
Chest circumference (CC)	0.85	0.722	<0.0001
Head circumference (HC)	0.825	0.680	<0.0001
Foot length (FL)	0.746	0.557	<0.0001

### Linear regression analysis

Three linear regression models for CC, HC and FL as independent variables and birth weight as dependent variable were created. The highest coefficient of correlation (R) and coefficient of determination (R^2^) were associated with CC followed by head circumferences and then foot length. All the correlations were significant at *p* < 0.001. CC had the lowest standard error of the estimate (SEE) and it had a higher coefficient of determination (R^2^) when compared with HC (0.722 vs. 0.680). FL gave a coefficient of determination (R^2^) of 0.557 which is the least.

**Fig. 1:**
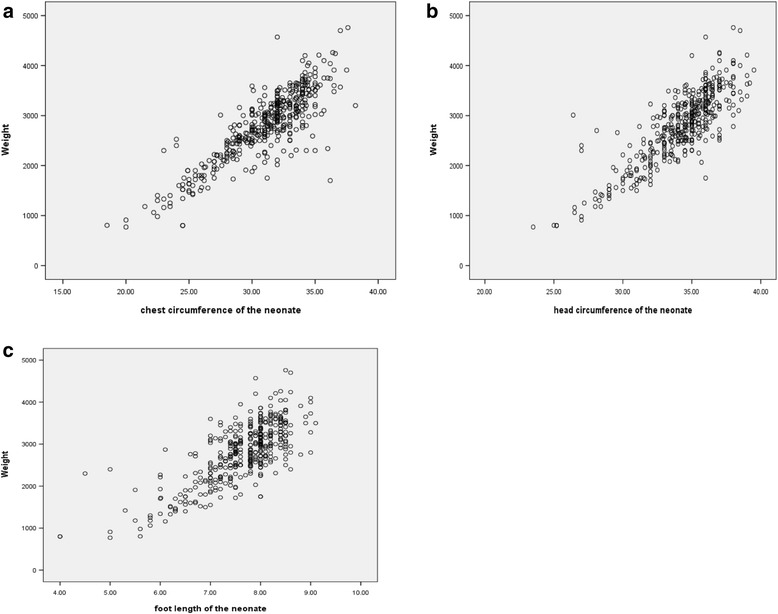
Scatter plots of the different anthropometric parameters for birth weight. **a**: Scatter plot of birth weight (g) on chest circumference (cm) for neonates. **b**: Scatter plot of birth weight (g) on head circumference for neonates. **c**: Scatter plot of birth weight (g) on foot length for neonates

Three scatter plot graphs were created, each representing CC, HC, and FL for the newborns (Fig. 1a–c). The positive gradients of the scatter plot diagrams show that there is a linear and positive relationship between all the anthropometric measurements and birth weight with the highest coefficient of determination (R^2^) being associated with CC.

### Receiver operating characteristic (ROC) curve analysis for cut-off point determination

**Fig. 2:**
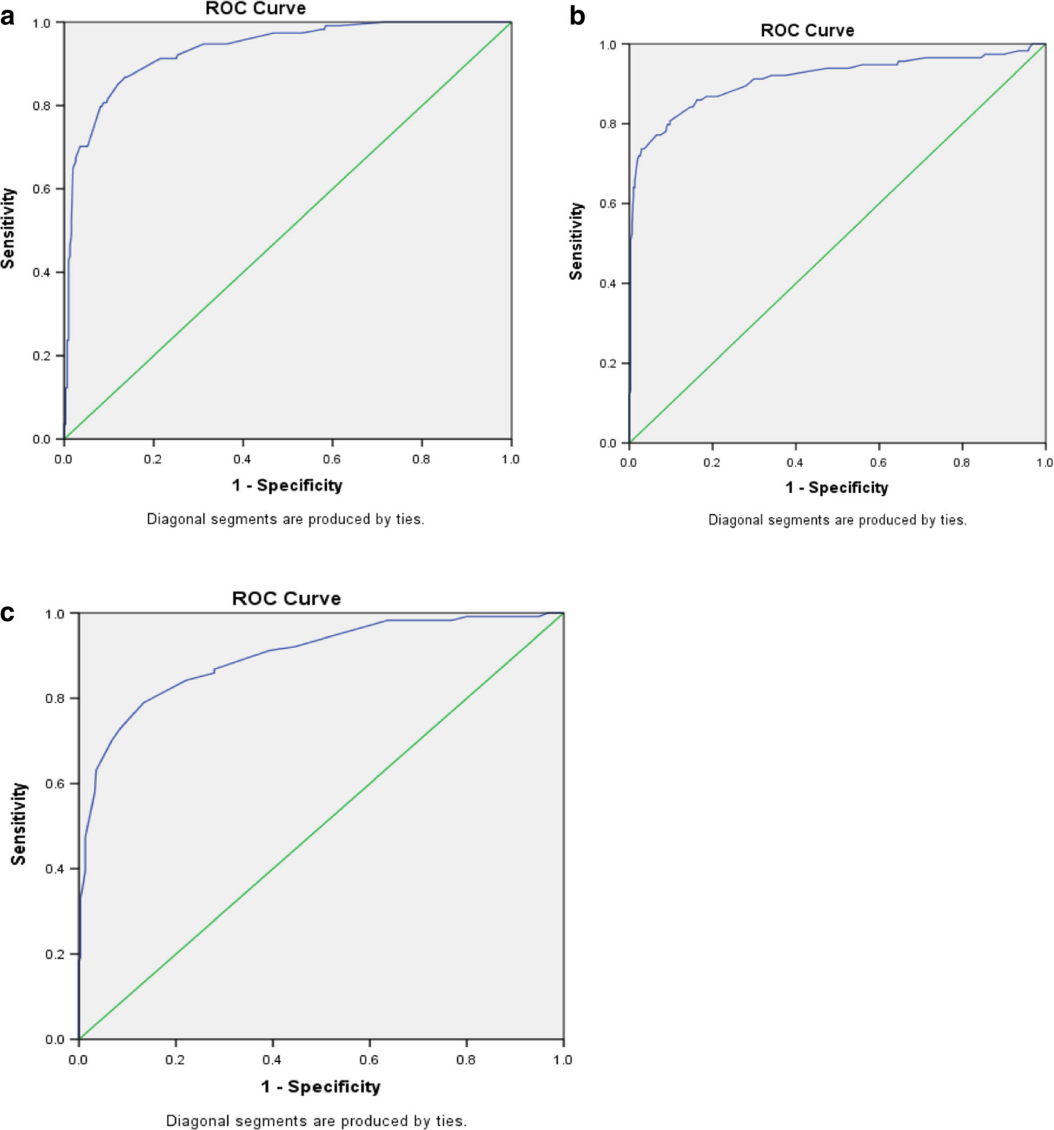
ROC curve for surrogate anthropometies. **a**. CC as a surrogate for birth weigth <2500gm, **b**. HC as a surrogate for birth weigth <2500gm, **c**. FL as a surrogate for birth weigth <2500gm

The corresponding ROC curves for CC, HC and FL as surrogates for birth weight less than 2500 g are shown in Fig. 2a–c. ROC analysis to test how well the different measures predict LBW showed that HC and CC have comparable area under the curve (AUC) 0.933 and 0.912 respectively (Table 5). For three of the anthropometric measures, sensitivity and specificity at every value were calculated then the possible operational cut-off points were determined by taking the highest average of sensitivity and specificity. Then the predictive performance of each of the cut off points was calculated as outlined on Table 6.

**Table 5:** AUC analysis for discrimination of birth weights below 2500 g

Parameter	AUC	95% CI
Head circumference	0.933	0.908 – 0.960
Chest circumference	0.912	0.873 – 0.950
Foot length	0.897	0.861 – 0.934

**Table 6:** Predictive performance of selected median cut-off points of CC, OFC and FL indices for birth weight <2500 g

Cutoff points	Sensitivity (%)	Specificity (%)	PPV (%)	NPV (%)
CC(30.15 cm)	84.2	85.4	67	93.5
HC(33.25 cm)	80.7	90	77	92.7
FL(7.45 cm)	78.9	86.7	72	91

*PPV* positive predictive value, *NPV* negative predictive value

For CC, the identified cut-off point was 30.15 cm with a sensitivity of 84.2% and 85.4% specificity. The optimal cutoff point for HC was 33.25 cm with a sensitivity of 80.7% and 90% specificity. With respect to FL the optimal cutoff point was 7.35 cm with 72.8% sensitivity and 91.6% specificity. With this CC is the most sensitive measurement followed by HC which has the highest positive predictive value (Table 6).

## Discussion

In this study, the prevalence of LBW was 27%; of this proportion of LBW among females were 57.9%. The mean birth weight was 2807 ± 691.82 g. Prematurity accounted for 74(17.5%) of the enrolled neonates. The LBW prevalence is a lot higher than the estimated national average which is 14% [7]. But studies show that the incidence is on an increasing trend among health institution deliveries for example in Gondar, Ethiopia the prevalence is 17.4% [15] and in the south western part of the country, Jimma it is 22.5% [7]. The higher prevalence of LBW can be attributed to the fact that the study was carried out in a referral hospital where high risk pregnancies that may result in preterm delivery are managed and where sick newborns are admitted.

Birth weight has been and is still an important screening tool for detecting newborns with LBW. Because of unavailability or inaccurate weighing scales and higher incidence of home deliveries measuring weight and detecting LBW neonates in developing countries is difficult [16]. This has led to the need for alternative measurements to assess newborns. A measuring tape is easily portable and can be used by community based health workers when they are notified of a delivery in a community under their catchment.

In the current study involving babies admitted to NICU and those born in Ayder Referral Hospital, birth weight correlated very strongly with the anthropometric variables of CC and HC. CC and HC demonstrated the best correlation with birth weight (*r* = 0.85 and AUC = 0.91) and (*r* = 0.82 and AUC = 0.93) respectively. Other analyses (sensitivity, specificity, PPV and NPV) also indicate that they are the two most appropriate measurements for identifying LBW in the study area. Though HC is not found to be the best surrogate anthropometric tool in most of the studies reviewed, the current finding is similar with a study done in Pokhara among Nepalese newborns. The study found the optimal cut-points for head circumference and chest circumference to identify LBW newborns to be 33.5 cm and 30.8 cm respectively [9]. This is comparable with the cut off points found in this study (33.25 for HC and 30.15 for CC). This is also true in a study done in newborns in Pelotas, Brazil which shows HC with cutoff point of 33 cm and CC with 31 cm to be good surrogates [6].

Foot length had the least correlation among all the parameters analyzed in the current study (*r* = 0.74) and was found to be less sensitive as well (72.8%). But in a study done in Uganda it correlated strongly with birth weight (*r* = 0.76 and AUC = 0.97) and had 94% sensitivity and 83% specificity in detecting LBW [10]. The study also implied that the measurement of foot length exposes the baby less which decreases the risk of hypothermia and is technically easier which are both important points that mandates further look into this anthropometric measurement for it is the least studied as compared to the others. Other studies also found less studied surrogates to be best in detecting LBW neonates; a study done in south eastern Nigeria comparing length, head circumference, mid arm circumference and maximum thigh circumference found that maximum thigh circumference to be the best surrogate for the detection of LBW neonates [17].

CC is the best surrogate anthropometric measurement in this study. This is similar to findings in Bangladesh [14]. This is also true with findings from studies done in Pokhara [9] which have reported good correlation between CC and birth weight. The high coefficient of correlation in the current study further reinforces the recommendation of the WHO collaborative study to use CC as an alternative measurement for detection of low birth weight [16].

The cut-off point for LBW in the current study was similar to value obtained In India [12], which was 30 cm. The cutoffs in studies done in Nepal and Bangladesh were 30.3 cm and 30.5 cm respectively [14, 18] but the value is lower than Egypt and Brazil which in both cases is 31 cm [6, 8]. The findings of the current study and other studies from both Africa [8, 10, 11] and Asia [12, 14] fall between a range of 30 cm and 31.0 cm.

Though both CC and HC have good correlation with birth weight, we recommend the use of chest rather than head circumference as a surrogate for birth weight for different reasons. CC is simpler to measure because the identification of the nipple line is easier; this makes measurement more operationally feasible than that of head circumference. Unlike on the head there are no significant soft tissue changes that can happen by the delivery process on the chest. The variations in the degree of molding and edema may make HC less accurate. Moreover, the use of head circumference alone can potentially miss a number of LBW infants who have normal head circumference measures but are LBW such as in the cases of asymmetric intrauterine growth restriction or the reverse with normal birth weight but reduced head circumference as in the case of chromosomal abnormalities and prenatal infections that may result in microcephly.

Therefore a tool which is easy to use with low cost and risk to identify small babies could be an effective intervention to save the lives of neonates. This can be implemented by the development of color coded tapes which can be used by trained community based health workers (like in Ethiopia health extension workers) working at remote areas where weighing scales might not be readily available.

### Study limitations

The fact that more than one data collectors were used may have an effect on the quality of data collected due to inter observer difference. But this was minimized by training the data collectors. In this study the measurements were done by health professionals but the tool will be used by health extension workers and their skill is likely to be different.

## Conclusion

Chest circumference and head circumference with cut off points 30.15 cm and 33.25 cm respectively are better surrogates for detecting LBW neonates. Of the two tools this study suggests that chest circumference is the most appropriate predictor given its high correlation and sensitivity in detecting LBW babies.

Neonates who fall below the stated cut off point should be considered as high risk and referred to health institutions where they can get better service and chance of survival.

## Abbreviations

ARH: Ayder referral hospital
AUC: Area under the curve
CC: Chest circumference
CI: Confidence interval
FL: Foot length
HC: Head circumference
MUAC: Mid upper arm circumference
NICU: Neonatal intensive care unit
NPV: Negative predictive value
PPV: Positive predictive value
ROC: Receiver operating characteristics
WHO: World Health Organization
